# A cross-sectional assessment of diabetes self-management, education and support needs of Syrian refugee patients living with diabetes in Bekaa Valley Lebanon

**DOI:** 10.1186/s13031-018-0174-9

**Published:** 2018-09-12

**Authors:** James A. Elliott, Debashish Das, Philippe Cavailler, Fabien Schneider, Maya Shah, Annette Ravaud, Maria Lightowler, Philippa Boulle

**Affiliations:** 1 Médecins Sans Frontières/Doctors Without Borders Canada, 551 Adelaide St W, Toronto, ON M5V 0N8 Canada; 2 Médecins Sans Frontières, Rue de Lausanne 78, Geneva, 1202 Switzerland; 3 Karolinska Institute, Stockholm, Sweden; 4 T1International, Cheltenham, UK

**Keywords:** Diabetes, Self-management, Global health, Refugee health, Non-communicable disease, Chronic disease, Syrian refugees, Lebanon

## Abstract

**Background:**

Patients with diabetes require knowledge and skills to self-manage their disease, a challenging aspect of treatment that is difficult to address in humanitarian settings. Due to the lack of literature and experience regarding diabetes self-management, education and support (DSMES) in refugee populations, Medecins Sans Frontieres (MSF) undertook a DSMES survey in a cohort of diabetes patients seen in their primary health care program in Lebanon.

**Methods:**

Structured interviews were conducted with diabetes patients in three primary care clinics between January and February 2015. Scores (0–10) were calculated to measure diabetes core knowledge in each patient (the DSMES score). Awareness of long-term complications and educational preferences were also assessed. Analyses were conducted using Stata software, version 14.1 (StataCorp). Simple and multiple linear regression models were used to determine associations between various patient factors and the DSMES Score.

**Results:**

A total of 292 patients were surveyed. Of these, 92% had type 2 diabetes and most (70%) had been diagnosed prior to the Syrian conflict. The mean DSMES score was 6/10. Having secondary education, previous diabetes education, a ‘diabetes confidant’, and insulin use were each associated with a higher DSMES Score. Lower scores were significantly more likely to be seen in participants with increasing age and in patients who were diagnosed during the Syrian conflict. Long-term complications of diabetes most commonly known by patients were vision related complications (68% of patients), foot ulcers (39%), and kidney failure (38%). When asked about the previous Ramadan, 56% of patients stated that they undertook a full fast, including patients with type 1 diabetes. Individual and group lessons were preferred by more patients than written, SMS, telephone or internet-based educational delivery models.

**Conclusions:**

DSMES should be patient and context appropriate. The variety and complexities of humanitarian settings provide particular challenges to its appropriate provision. Understanding patient baseline DSMES levels and needs provides a useful basis for humanitarian organizations seeking to provide diabetes care.

**Electronic supplementary material:**

The online version of this article (10.1186/s13031-018-0174-9) contains supplementary material, which is available to authorized users.

## Background

Diabetes is a common reason of medical consultation for Syrian refugees [1, 2]. The prevalence of diabetes among Syrian adults before the ongoing crisis was estimated to be 9% [3]. About 5,654,807 Syrians have fled to other countries in the region and registered as refugees as of April 2018. In 2014, the time of this study, the Bekaa Valley of Lebanon was the place of residence of 410,000 Syrian refugees registered with the United Nations High Commissioner for Refugees (UNHCR), 35% of the total number of Syrian refugees registered in Lebanon [4]. Many Syrian refugees in Lebanon face poverty and food insecurity, complicating the management of diabetes [5].

Médecins Sans Frontières (MSF) is an international, independent, medical humanitarian organization that responds to emergency situations and provides medical care to people in need affected by conflict, epidemics, natural disasters, and exclusion from healthcare [6]. MSF began an emergency medical intervention for Syrian refugees in Bekaa Valley, Lebanon in February 2012, including diabetes management.

### Diabetes care in MSF clinics in Bekaa Valley Lebanon

Bekaa Valley is an agricultural region in Lebanon that directly borders western Syria. The region is predominately rural, with some small cities. The vast majority of residents are culturally and linguistically Arabic, and belong to Shiite, Sunni and various Christian religious denominations. MSF ran four primary care clinics in the Bekaa Valley at the time of this study, providing primary health care as well as diabetes and other non-communicable disease (NCD) management, mental health support, and mother and child health services. Diabetes care consisted of provision of free medications, including oral hypoglycaemic agents (OHAs) and human insulins, distribution of a limited number of blood glucose meters and test strips (primarily to patients taking insulin), nursing care, some patient education in the form of pamphlets and community health worker group lessons, and limited laboratory investigations. General practitioners provided the routine care with support from nurses. Clinics scheduled patients with NCDs for appointments on specific days of the week. Most patients visited the clinic at least once per month. At the end of 2014, a total of 1030 patients diagnosed with diabetes were in active follow up in MSF primary care clinics in Bekaa Valley. Of these, 51 had type 1 diabetes and 979 had type 2 diabetes.

### Diabetes self-management education and support: Important in all contexts

It is critical for people with diabetes to understand how to self-manage their condition [7]. Patient-related factors have the largest impact on blood glucose control [8]. Therefore patients with diabetes must be supported to monitor and control factors that influence blood glucose to the best of their abilities in order to decrease the frequency of hyperglycaemia and hypoglycaemia and improve long-term outcomes. These factors include but are not limited to diet, medication, and physical activity [9, 10]. As blood glucose volatility increases, so do the chances of severe complications, both acute and chronic [11–13].

Diabetes Self-Management Education and Support (DSMES) refers to the education and support patients need for diabetes self-management. DSMES interventions focus on healthy eating, physical activity, prevention and management of hypo−/hyperglycemia, prevention and surveillance of complications, and medication management, including insulin dose titration. DSMES encourages active patient participation in self-monitoring and decision making. Research has shown DSMES is a crucial determinant of health and quality of life for people living with diabetes [14, 15], and it has been shown to be effective in low, middle and high income contexts [16, 17].

### NCD and DSMES research in comparable populations

NCD research in both Jordan and Lebanon has examined the situation among Syrian refugees. In Jordan staff training, context-specific patient considerations, rapport with patients, and an understanding of the psycho-social-occupational context of patients were found to be enablers of effective NCD program implementation. Work by Gammouh et al. who found that newly diagnosed chronic diseases and lack of medications significantly contributed to depression of Syrian refugees living in Jordan. Thus the need for contextual understanding seems paramount. In Lebanon, research by Sethi et al. found volunteer refugee health workers effective in implementing community-based primary health activities for Syrian refugees living with NCDs, suggesting this may be a useful strategy where resources available for NCD care are limited [18–21].

Some DSMES studies exist in comparable refugee settings. A study of Syrian women living with diabetes before the Syrian conflict showed inadequate patient education and poor patient knowledge [22]. In higher income Arab countries DSMES research is more common. Omani patients were found to have a lack of self-management knowledge and limited awareness of long-term diabetes complications [23]. A study in the United Arab Emirates revealed similar findings [24].

Structured DSMES programs, which have used a variety of modalities including face-to-face interviews, telephone based interventions, written educational material, classes and other means, have been shown to be effective for populations in the Middle East region [25]. Different studies have demonstrated improved patient knowledge and self-care [26], and improved glycaemic control and quality of life [27]. .Iranian qualitative research has also highlighted the importance of addressing context specific socio-cultural factors in order to achieve optimal diabetes control [28]. These included stressors associated with the costs of treating diabetes, as well as expectations of the family and the health system regarding the behaviour, adherence and perceived burden of the patient living with diabetes. The literature also highlights DSMES innovations. In Iraq, a small but statistically significant trial used an SMS (text-message) education program to increase patient knowledge and reduce HbA1c, an indicator of overall blood glucose control [29].

At the time of this study there was no peer reviewed published research on DSMES of Syrian refugees living with diabetes in Lebanon. The aim of this study was to conduct an assessment of patient needs related to DSMES in order to design adapted interventions that would improve patient self-care, coping skills, knowledge, health and quality of life.

## Methods

### Study design

A survey of patients receiving diabetes care in MSF Bekaa Valley clinics was performed. The sample frame was adult refugees of the Syrian conflict seeking care in 3 of the 4 facilities operated by MSF located in the Bekaa Valley region of Lebanon with type 1, type 2 or an indeterminate type of diabetes. One facility had to be excluded for security reasons. Content and design of the survey instrument was based on previous research [27, 28, 30, 31]. The initial version of the survey was piloted with 12 patients in one of the Bekaa Valley facilities, resulting in constructive adjustments. The final version of the survey included demographic, social, emotional, behavioural, diabetes history and educational needs/preferences components (Additional file 1: Appendix S1).

### Inclusion and exclusion criteria

All adult Syrian refugees seeking care for diabetes in MSF clinics in Bekaa Valley Lebanon were eligible for the study. Patients aged under 18 years of age, those with gestational diabetes, and those who refused were excluded.

### Sample size calculation

At the time approximately 1000 patients with diabetes were receiving care from MSF in Bekaa Valley Lebanon. Anticipating future research, this study aimed to detect a change of at least 20% improvement (or deterioration) in the DSMES score variable from baseline. Assuming an alpha error of 5%, and a statistical power of 80%; 107 individuals represent the minimum sample size for the detection of a 20% variation of this variable. Anticipating a 5% refusal rate and a 30% loss to follow-up, and planning on follow-up investigations, the final requirement for sample size was then of 146 individuals.

### Survey administration

MSF recruited two data collectors (a nurse and a community member) who had been previously trained on survey administration. They were trained on the survey, and on basic diabetes education e.g. signs and symptoms of hyper/hypoglycaemia. The surveyors were instructed not to lead participants, not to give clues, and not to mime the correct answers. Data collectors then verbally administered the survey in Arabic, and in English if requested, in a private environment within the MSF clinic where they were receiving care. Data collection took place over a period of 21 days, from January 20th to February 12th 2015. All patients meeting inclusion criteria were opportunistically sampled in the clinics on days a data collector was present in clinic. Patients were asked to give written consent after the study aims and their right to refuse with no consequence to future treatment was explained to them. Patients who gave written informed consent were then interviewed in a private location in the clinic by one of the data collectors. Data collectors recorded patient responses to open questions verbatim (exactly as spoken). After the survey, data collectors gave patients a short educational session to address key weaknesses in diabetes knowledge that were displayed, and alerted the clinical staff if the patient had life-threatening misconceptions (such as believing insulin corrected hypoglycaemia).

### DSMES scores

A measure of patient DSMES (the DSMES score) was created through five open-ended core questions. The scoring system was based on that used by Elliott et al. 2013 [23], and modified by the study team to be more context appropriate These questions gauged key areas of knowledge for patient self-management of their diabetes: recognition of hyperglycaemia, response to hyperglycaemia, recognition of hypoglycaemia, response to hypoglycaemia, and knowledge of strategies to stabilize blood glucose levels. Two investigators (JE, PB) developed the scoring rubric and then acted independently as evaluators in its application (Additional file 2: Appendix S2). In some cases context informed the appropriateness of responses. For example if a respondent said “I eat bread” in response to having hypoglycemia, this perhaps would not be recommend in a high income setting, where faster-acting glucose sources like juice may be more preferable. However in this context bread may be the only source of carbohydrate on hand and thus an appropriate response. Differences in evaluation were resolved through discussion. The sum of the five core questions formed the DSMES score for each patient. The maximum a patient could score was 10/10, the minimum was 0/10.

### Data analysis

Verbatim responses were grouped e.g. ‘*eye disease*’ and ‘*bleeding in the eyes’* were classified as ‘*vision related complications’*. Analyses were conducted using Stata software, version 14.1 (StataCorp). Linear regression model was used to determine the factors associated with DSMES Score of patients. In univariate analysis, each of the following variables were considered: age, sex, duration and type of diabetes, level of education, previous diabetes education, years with known diabetes, Ramadan fasting, having a confidant for diabetes, oral medication or insulin use, self-measurement of blood glucose and diabetes diagnosed during Syrian conflict. All the variables which were significant at 5% level in univariate analysis were considered for multivariable analysis. A multivariable regression model was then constructed using all the variables identified from univariate analysis. ‘Sex’ was retained in the multivariable analysis regardless of statistical significance as it is considered as an important demographic characteristic. The aim was to identify independent predictors of DSMES Score and hence variables which were not significant in the multivariable model in the presence of other variables were excluded using backwards elimination. The influence of removing the non-significant variables from the multivariable model was further gauged upon by their effect on the coefficient and statistical significance of other variables retained in the final model. Effect sizes were deemed as being statistically significant if the associated *p*-value from the Wald’s test of the regression coefficients were < 0.05. Furthermore, the normality of residuals after running the regression analysis was checked and the residuals were close to a normal distribution. An assessment of multi-collinearity showed that multi-collinearity wasn’t an issue in the regression analysis.

### Ethics, consent, permissions

Patient participation in this study was voluntary. A note explaining the study rationale and procedures, including the right to refuse participation with no consequence to their medical care, was read to the patient in their choice of Arabic or English. An opportunity to ask any questions or queries was given to prospective participants. If consent was not given, the reason for refusal was noted. All study participants included in this study gave written informed consent. These records are kept in the MSF Swiss Beirut coordination office under lock and key. All participant data was kept de-identified, and confidential. The Médecins Sans Frontières Ethics Review Board approved the study protocol (ID #1423). At all times this study was performed in accordance with the principles of the Declaration of Helsinki [32].

## Results

### Demographics

A total of *n* = 295 patients were approached and *n* = 292 patients with diabetes were enrolled in the survey (Tables 1 and 2). There were three recorded refusals, all due to lack of patient time. Patients’ ages ranged from 18 to 84 years, with a median of 54. Nearly half (42%) of those had not completed any formal education; 12% had education beyond primary school. Over half of the patients surveyed (63%) provided a mobile phone number.

**Table 1:** Demographics of patients with diabetes in MSF clinics in Bekaa Valley, Lebanon

Characteristic	Diabetes Study Group (*N* = 292)
Sex, No. (%)	
Male	112 (38)
Female	180 (62)
Age, years	
Mean (SD)	54 (13)
Age Group, No. (%)	
18–29	15 (5)
30–39	17 (6)
40–49	53 (18)
50–59	101 (34)
60–69	78 (27)
70+	28 (10)
Level of Education, No. (%)	
None	118 (40)
Primary	131 (45)
Secondary	23 (8)
Post-secondary	10 (3)
Unknown	10 (3)
Recruitment Centres, No. (%)	
1. Baalbek PHC	70 (24)
2. Hermal PHC	12 (4)
3. Majdal Anjar	210 (72)

Abbreviations: *SD* standard deviation, *PHC* primary health centre

**Table 2:** Diabetes characteristics of patients with diabetes in MSF clinics in Bekaa Valley, Lebanon

Characteristic	Diabetes Study Group (*N* = 292)
Type of Diabetes, No. (%)	
Type 1	22 (8)
Type 2	270 (92)
Duration of Diabetes, years	
Median (Range)	8 (0–30)
Years with Known Diabetes, No. (%)	
1 or less	23 (8)
2–3	33 (11)
4–5	56 (19)
6–9	60 (21)
10 or more	119 (41)
Diabetes Diagnosed During Syrian Conflict, No. (%)	
No	203 (70)
Yes	88 (30)
Previous Diabetes Education, No. (%)	
Received	149 (51)
None	143 (49)
Medication Use, No. (%)	
Insulin only	34 (12)
Oral only	216 (74)
Both insulin and oral	39 (13)
No medication	3 (01)
Self-measurement of Blood Glucose, No. (%)	
No	229 (78)
Yes	63 (22)

### Diabetes characteristics (Table 2)

Most patients (92%) had type 2 diabetes. Median duration since diabetes diagnosis was 8 years; the longest known duration was 30 years. Self-monitoring of blood glucose (SMBG) was more common among type 1 diabetes patients than type 2 diabetes patients (73% vs. 17%, *p* = *p* < 0.001), likely influenced by the limited free distribution by MSF of blood glucose meters and testing strips to some patients taking insulin. Most patients reported complete adherence to diabetes medications during the previous 7 days (88%, *n* = 256).

### Social and self-reported health findings

The majority of patients reported eating two (36%, *n* = 105) or three times (52%, *n* = 153) per day. Around half (54%, *n* = 159) said they had someone to talk to about diabetes. 53% (*n* = 154) self-reported their health as ‘*good*’ on a 5 point scale ranging from poor (score = 1) to excellent (score = 5). Ramadan fasting was highly prevalent. Patients with type 2 diabetes were significantly more likely to have undertaken a full fast than patients with type 1 diabetes (60% vs 14%, *p* < 0.001). For patients with type 1 diabetes, *n* = 3 (14%) claimed to have fully fasted, and *n* = 4 (18%) stated they held a partial fast or had tried but had to break their fast. In total 40% of those using insulin fasted, but were less likely to have a full fast than those not on insulin (29% vs. 65%, *p* < 0.001).

### Diabetes knowledge

**Fig. 1:**
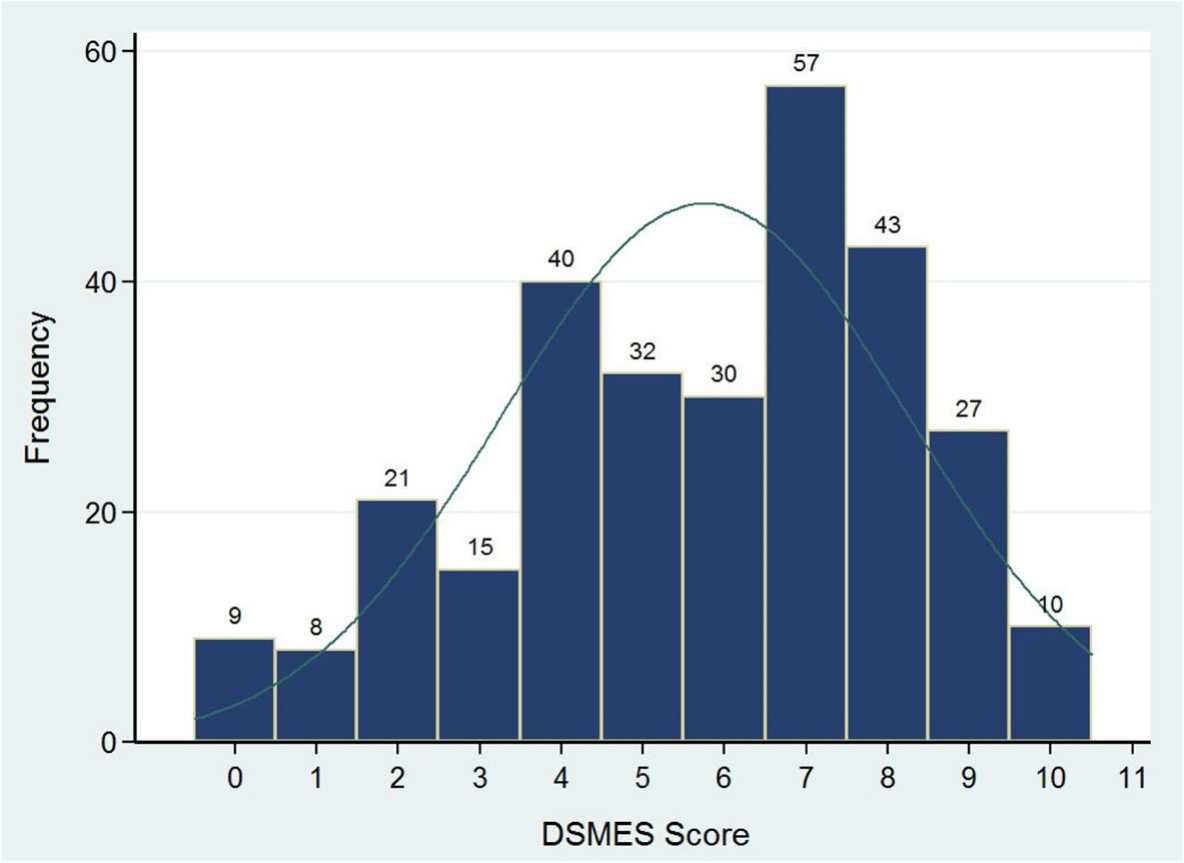
Distribution of DSMES scores (higher is better) of patients with diabetes surveyed in MSF clinics in Bekaa Valley, Lebanon; Jan – Feb 2015

The distribution of DSMES score is shown in Fig. 1. Mean score was 6/10 (IQR 4–8), with 10 patients receiving the maximum score, and nine the minimum. Inability to name a sign or symptom of hypoglycaemia (34%, *n* = 98) and respond to hypoglycemia (35%, *n* = 102) were more common than inability to name a sign or symptom of hyperglycaemia (8%, *n* = 24) and respond to hyperglycaemia (13.7%, *n* = 40). Around 1 in 5 patients (21%, *n* = 60) could not mention a strategy to normalize blood glucose.

Regarding knowledge of long-term diabetes complications, vision-related complications were the most well-known (68%, *n* = 197). This was followed by foot ulcers (39%, *n* = 115) and kidney problems (38%, *n* = 110). Sex-related issues (2%, n = 6) were mentioned exclusively by men and only to the male data collector. 22% (63) of patients were unable to mention a complication.

### Education needs and preferences

When asked for which topics patients needed more information, diet (90%, *n* = 263), diabetes complications (82%, *n* = 238) and medications (71%, *n* = 208) were the most commonly mentioned. Other topics mentioned included hypo/hyperglycaemia (55%, *n* = 159), exercise (43%, *n* = 125) and stress (41%, *n* = 120). The majority of the patients reported that they were comfortable receiving diabetes education from doctors (97%, *n* = 283) or nurses (78%, *n* = 229). By comparison, fewer patients were comfortable receiving education from dieticians (50%, *n* = 146), community health workers (47%, *n* = 136) or fellow patients (46%, *n* = 133). The most preferred educational formats were group lessons (94%, *n* = 274) and individual lessons (81%, *n* = 237). These eclipsed written materials (7%, *n* = 19), internet formats like Twitter or email (4%, n = 12), telephone calls (2%, *n* = 7) and SMS/text message (2%, *n* = 5).

### Correlations with DSMES score

**Fig. 2:**
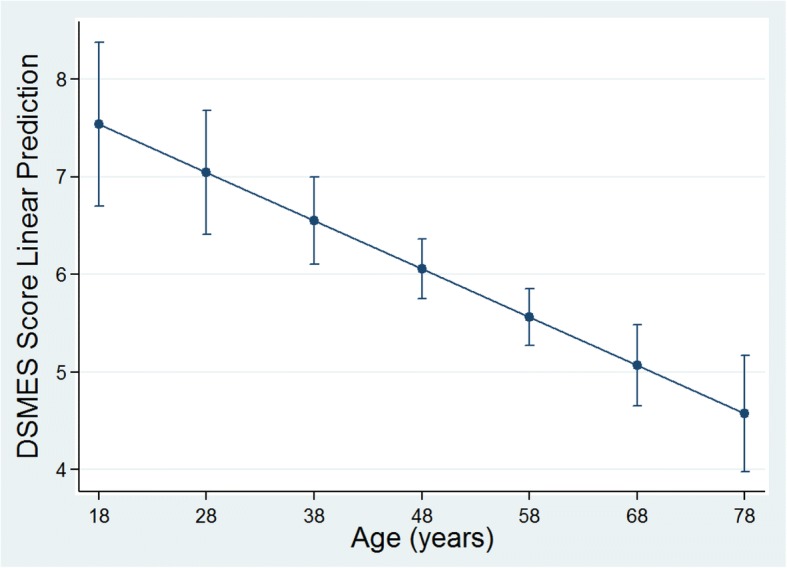
Adjusted predictions of mean DSMES score in relation to age with 95% confidence intervals

In the univariate analysis, there was a negative linear relationship between age and DSMES Score (Fig. 2 and Table 3). For an increase in age of the participant by one year, there was a decrease in the value of average DSMES score of 0.05 (*p* = < 0.001). However, duration of diabetes, secondary education, previous diabetes education, having a confidant for diabetes, insulin use, self-measurement of blood glucose were independently positively associated with DSMES Score.

**Table 3:** Univariate analysis of factors associated with DSMES Score of patients with diabetes in MSF clinics in Bekaa Valley, Lebanon

Variable	Coefficient	95% CI	*p*-value
Age	−0.05	− 0.07, − 0.03	*< 0.001*
Duration of Diabetes	0.13	0.08, 0.17	*< 0.001*
Sex			
Male	Base category		
Female	−0.18	− 0.77, 0.41	0.555
Type of Diabetes			
Type 1	Base category		
Type 2	−2.33	−3.38, −1.27	*< 0.001*
Level of Education			
None	Base category		
Primary	0.65	0.04, 1.26	*0.037*
Secondary	2.12	1.03, 3.21	*< 0.001*
Post-secondary	1.27	−0.31, 2.85	0.114
Previous Diabetes Education			
None	Base category		
Received	1.28	0.72, 1.83	*< 0.001*
Years with Known Diabetes			
1 or less	Base category		
2–3	0.46	−0.78, 1.70	0.465
4–5	0.58	−0.56, 1.71	0.318
6–9	2.23	1.10, 3.35	*< 0.001*
10 or more	2.36	1.31, 3.40	*< 0.001*
Ramadan Fasting			
No	Base category		
Yes^a^	− 0.78	−1.42, − 0.15	*0.016*
Having A Confidant for Diabetes			
No	Base category		
Yes	1.07	0.51, 1.64	*< 0.001*
Insulin Use			
No	Base category		
Yes	2.19	1.57, 2.80	*< 0.001*
Oral Medication Use			
No	Base category		
Yes	−2.12	−2.96, −1.29	*< 0.001*
Self-measurement of Blood Glucose			
No	Base category		
Yes	1.14	0.45, 1.83	*0.001*
Diabetes Diagnosed During Syrian Conflict			
No	Base category		
Yes	−2.02	−2.60, −1.44	*< 0.001*

^a^ Combination of full and partial fasting

*p*-value are statistically significant

After controlling for factors in the multivariable regression model, secondary education, previous diabetes education, having a confidant for diabetes, and insulin intake remained statistically significant and associated with higher average DSMES Score (Table 4). Participants with increasing age and diabetes diagnosed during the Syrian conflict were significantly more likely to have lower DSMES Score.

**Table 4:** Multivariable linear regression model for factors influencing DSMES Score of patients with diabetes in MSF clinics in Bekaa Valley, Lebanon

Variable	Adjusted Coefficient	95% CI	*p*-value
Age	−0.04	−0.06, −0.01	*0.003*
Duration of Diabetes	0.03	−0.03, 0.09	0.331
Sex, Female	−0.11	− 0.64, 0.43	0.696
Level of Education			
Primary	0.03	−0.55, 0.60	0.931
Secondary	1.03	0.03, 2.03	*0.043*
Post-secondary	0.1	−1.31, 1.51	0.892
Receiving Previous Diabetes Education	0.49	−0.02. 1.00	0.059
Ramadan Fasting	0.39	−0.21, 0.99	0.205
Having a Confidant for Diabetes	0.8	0.27, 1.33	*0.003*
Insulin Use	1.43	0.66, 2.19	*< 0.001*
Diabetes Diagnosed During Syrian Conflict	−1.56	−2.27, −0.85	*< 0.001*

*p*-value are statistically significant

## Discussion

This study is the first known published assessment of diabetes core knowledge and self-management in Syrian refugees living with diabetes in Lebanon. Many patients were unable to express core diabetes knowledge needed for self-monitoring and treatment. Particularly troubling was the inability of some patients to mention a way to recognize hypoglycaemia (34%, *n* = 98) and respond to hypoglycemia (35%, *n* = 102). This has implications for both acute and chronic morbidity, and potentially mortality. The high number of patients who did not know how to recognize and/or respond to hypoglycaemia raises questions around patient safety. Some associations with a higher DSMES score were expected, such as having had previous diabetes education or a secondary education. The stark difference observed in patients diagnosed after the onset of Syrian crisis suggests conflict and displacement have had a detrimental impact on DSMES.

Patients using insulin were also more likely to have a higher score. This may due to more attention being paid to education for insulin users. Having a confidant for diabetes was an interesting factor that was positively associated with a higher DSMES score, and interventions utilizing involvement of a family member or other patient supporter merit further attention. Family support interventions may be especially beneficial given the tight social bonds of this population. Research shows these interventions may improve metabolic and behavioural outcomes in both type 1 and type 2 diabetes [33, 34].

Many patients fasted or attempted to fast during the previous Ramadan, in line with rates seen in Muslim populations in the region [35]. The precise reasons for fasting were not measured via the survey tool, but religious, cultural and family-linked factors are probable. Organizations treating Muslim patients should prepare for Ramadan through provider training and patient-focused activities like practice fasts, temporary medication regimes and setting pre-determined circumstances when the fast should be broken [36]. We also note the guidelines recently released by Diabetes and Ramadan International Alliance for diabetes care during Ramadan [37], and the International Group for Diabetes and Ramadan, which call for focused patient education, regular glucose monitoring and adjustment of treatment regimens weeks prior to Ramadan [38].

The results of this study have shown that in this context, individual and group education was preferred by patients over written materials and electronic mediums. Preference for written materials scored surprisingly low, perhaps due to the low level of formal education among patients. Simple language, structure and the use of pictures is to be encouraged.

Dietary information was the most commonly requested subject. Diet is an important but difficult topic to address in contexts where adequate dietary intake is limited by financial constraints, which have only become more difficult due to funding cuts to the World Food Program [39]. Lastly, around 1 in 5 of the patients surveyed could not mention a single complication of diabetes, and including a discussion of possible diabetes complications (such as foot ulcers) is important, so that patients can self-monitor and know when to seek medical assistance, as well as understand the need for adherence.

Diabetes care is today a high priority for MSF [40]. MSF has developed clinical guidelines and tools for diabetes and comorbidity treatment. Simplified management is established in many settings, using general practitioners or clinical officers, and task shifting routine follow up care to nurses [41]. Diabetes self-management education and support is an essential element of this patient management regardless of context. In an emergency humanitarian crisis, where access to food, medication and supplies is challenging, it can be a matter of life or death [42]. Fostering self-management in patients can potentially improve patient self-care, coping skills, knowledge, health and quality of life, while simplifying clinical management e.g. patients being more aware of issues concerning their chronic disease(s) and how to communicate them to the provider, as well as rationalizing available resources.

However, DSMES provision remains challenging in the settings where MSF operates. Further, many of the self-management barriers which patients face are social determinants of health such as poverty, housing insecurity, unstable settings, and social isolation. MSF has however a long and innovative history of supporting self-management and patient education for other chronic diseases, especially TB and HIV/AIDS. Strategies used by MSF for other chronic diseases are being increasingly adapted to diabetes care [43], – methodology such as peer groups, pill clubs and task shifting – and this adaptation can be assisted by an assessment of the contextual factors [41]. Using a simple questionnaire such as this one to assess a baseline level of knowledge can provide a rapid understanding of core areas of knowledge, and allow a busy team to orient patient education to the important gaps.

### Limitations

The busiest MSF clinic in Bekaa Valley, located in Aarsal, was not included in this study due to security constraints [44]. HbA1c and other bio-markers were not incorporated into the study. A major limitation of this study is that the survey tool was made bespoke for this context and has not been validated against health outcomes or subject to an inter-rater reliability validation exercise. It also remains to be seen to what extent the benefits of DSMES demonstrated in other contexts are replicable.

## Conclusions

Humanitarian organizations treating patients with diabetes should anticipate the need to provide DSMES and tailor interventions based on the results of needs assessments. Assessing patient baseline DSMES levels and needs through a survey provides a useful basis for humanitarian organizations seeking to provide diabetes care. The results can be used to appropriately target DSMES interventions adapted to patients’ identified needs and preferences, as a component of a model of care adapted to the context.

## Additional files

Additional file 1: Appendix S1 Survey Instrument (English Version). (DOCX 105 kb)

Additional file 2: Appendix S2 DSMES Scoring Rubric. (DOCX 43 kb)

## Abbreviations

ANOVA: Analysis of variance
DSMES: Diabetes Self-Management, Education, and Support
HbA1c: Hemoglobin A1c
HIV/AIDS: Human Immunodeficiency Virus / Acquired Immune Deficiency Syndrome
IBM: International Business Machines Corporation
MSF: Médecins Sans Frontières (English = Doctors Without Borders)
NCD: Non-communicable Disease
OCG: Operational Centre Genève (MSF Swizterland)
OHA: Oral Hypoglycemic Agents
READ: Ramadan Education and Awareness in Diabetes
SMBG: Self-Monitoring of Blood Glucose
SMS: Short Message Service
TB: Tuberculosis
UNHCR: United Nations High Commissioner for Refugees
