# Supplementary material for: A cross-sectional assessment of diabetes self-management, education and support needs of Syrian refugee patients living with diabetes in Bekaa Valley Lebanon
Source: Confl Health. 2018 Sep 12;12:40. doi: 10.1186/s13031-018-0174-9 (PMC6134700; doi:10.1186/s13031-018-0174-9)
Supplement: Supplementary file 1 — Appendix S1 Survey Instrument (English Version). (DOCX 105 kb) [file 13031_2018_174_MOESM1_ESM.docx]

Appendix S1: Survey Instrument (English Version)

***MSF Diabetes Self-Management Education and Support Survey***

Patient # __________________ Mobile Telephone 1#:

Mobile Telephone 2#:

Sex: Female / Male Age: _________

Diabetes: a Type 1 Type 2 c) Unknown

1. Highest education completed: a) None b Primary c) Secondary Post-Secondary
2. How many years have you known that you have diabetes? ______

**I. Self-Care and Coping Component:**

3. Have you ever received any form of diabetes education? A Yes b No

1. How can you know if your blood sugar is low? Say as many ways as you can:
2. What do you do when your blood sugar is low? Say as many ways as you can:

1. How can you know if your blood sugar is high? Say as many ways as you can:
2. What do you do when your blood sugar is high? Say as many ways as you can:
3. What are ways you can keep your blood sugar from getting too high or low?

Say as many ways as you can:

1. Can you mention three long-term complications of diabetes? *List responses*

**1.**

**2.**

**3.**

**II. Social Component**

10. How many meals per day did you eat on average during the last week?

0 b 1 c 2 d 3 e 4 f 5 or more Cannot Recall

11. When Ramadan occurred this year and did you choose to fast?

Yes, fasted Partially/broke fast No, did not fast

12. Do you have a family member, friend or other confidant you can talk with about your diabetes and your treatment?

A Yes No

**III. Emotional Component**

1. In general, would you say that your health is excellent, very good, good, fair, or poor?

Excellent Very Good Good Fair Poor

**IV. Behavioural Component**

14. Do you take insulin(s)?

Yes b No

15. Do you take oral medication(s) for your diabetes?

Yes No

*If both Q14 and Q15 = no, skip to Q18.*

16. On how many of the last 7 days did you take your recommended diabetes medication(s)?

1 day 2 days 3 days 4 days 5 days 6 days 7 days

17. Do you check your blood sugar at home?

a) *If yes*, how many times per week: _________­­­

b) *If no*, why not? i) Unaffordable ii) Do not know how iii) Do not want to

Do not have glucometer machine

**V. Education Needs and Preferences**

18. What subjects, if any, do you need more information on?

*Read out options below, check all that apply and write in as needed*

1. Healthy eating/diabetic diet

Dealing with stress

1. Long-term diabetes complications/how to prevent them
2. Exercise
3. Medications

Recognizing/treating blood sugar ‘highs’ and ‘lows’

1. Other ___________________________
2. Do not need more information

19. From whom would you feel comfortable receiving diabetes information from?

*Read out options below, check all that apply and write in as needed*

Doctors

1. Nurses
2. Knowledgeable fellow patients living with diabetes
3. Community health workers

Dieticians

Other ___________________________

None/do not want diabetes information

20. In what formats would you like to receive diabetes information on?

*Read out options below, check all that apply and write in as needed*

Written materials, like books and brochures

1. Group lessons
2. Individual lessons

SMS text messages on mobile phone

1. Telephone calls

Internet/Twitter/Email

Other ___________________________

None/do not want diabetes information
