# Supplementary material for: A cross-sectional assessment of diabetes self-management, education and support needs of Syrian refugee patients living with diabetes in Bekaa Valley Lebanon
Source: Confl Health. 2018 Sep 12;12:40. doi: 10.1186/s13031-018-0174-9 (PMC6134700; doi:10.1186/s13031-018-0174-9)
Supplement: Supplementary file 2 — Appendix S2 DSMES Scoring Rubric. (DOCX 43 kb) [file 13031_2018_174_MOESM2_ESM.docx]

Appendix S2: DSMES Scoring Rubric

| **Question** | **Appropriate Answers** | **Scoring** |
| --- | --- | --- |
| **How can you tell if your blood sugar is low?** | Fatigue/sleepy/tired/drowsy, dizziness/loss of balance/vertigo, cold sweat, shaking/shivers, chills, slurred speaking, confusion/difficulty concentrating, lack of coordination/staggering gait, stressed/anxious/nervous feeling, excess hunger, headache, blurred vision, dizziness, abdominal pain, nausea, SMBG, fainting | *Two Correct = 2 One Correct = 1 None Correct = 0* |
| **What do you do when your blood sugar is low?** | Consume juice, raw sugar, honey, or any other quick way to ingest sugar orally | *Any Correct = 2 None Correct = 0* |
| **How can you tell if your blood sugar is high?** | Thirst/dry mouth/increased water intake, frequent urination, headaches, difficulty concentrating, blurred vision, fatigue/sleepy/tired/drowsy, SMBG, persistent vaginal and skin infections, slow-healing wounds, cold or insensitive feet, loss of hair on the lower extremities, erectile dysfunction, chronic constipation, wasting, anorexia, teeth and oral problems, bad breath, palpitations and shortness of breath (ketosis) | *Two Correct = 2 One Correct = 1 None Correct = 0* |
| **What do you do when your blood sugar is high?** | Drink water, inject insulin, take medication, exercise, and abstain from eating or cut back on portion size, SMBG, go to doctor* | *Two Correct = 2 One Correct = 1 None Correct = 0 *0 if sole response* |
| **What are some ways you can help keep your blood sugar from getting too high or low?** | Adjust medication and/or diet, take medication regularly, follow an exercise regime, eat at consistent times, keep track of food content and amounts, keep regular meal times, test blood sugar regularly, ask for advice from health care providers, stop smoking, relax/avoid stress | *Two Correct = 2 One Correct = 1 None Correct = 0* |
| *-1 per dangerous response, e.g. insulin for hypoglycaemia response* **Max Score = 10/10** | | |
